# Scanning Tunneling Microscopy Visualization of Polaron Charge Trapping by Hydroxyls on TiO_2_(110)

**DOI:** 10.1021/acs.jpcc.4c03751

**Published:** 2024-08-08

**Authors:** Chi-Ming Yim, Michael Allan, Chi Lun Pang, Geoff Thornton

**Affiliations:** † Department of Chemistry and London Centre for Nanotechnology, University College London, 20 Gordon Street, London WC1H 0AJ, U.K.; ‡ Tsung Dao Lee Institute and School of Physics and Astronomy, Shanghai Jiao Tong University, 1 Lisuo Road, Shanghai 201210, China

## Abstract

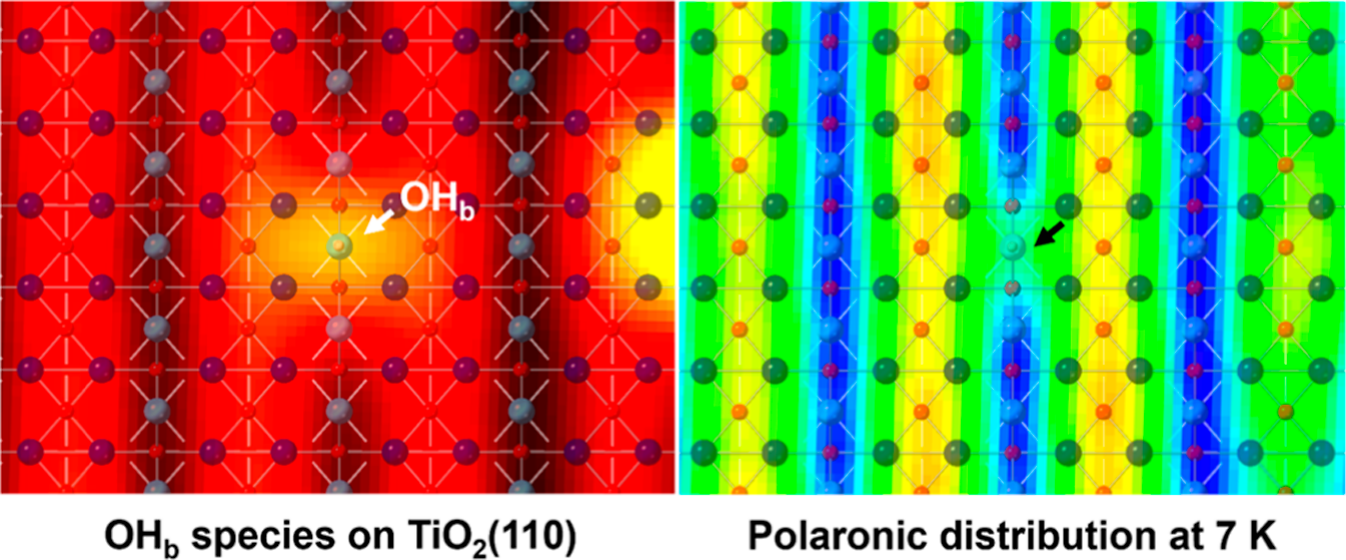

Using scanning tunneling microscopy (STM), we investigate the spatial distribution of the bridging hydroxyl (OH_b_) bound excess electrons on the rutile TiO_2_(110) surface and its temperature dependence. By performing simultaneously recorded empty and filled state imaging on single OH_b_s at different temperatures in STM, we determine that the spatial distribution of the OH_b_ bound excess electrons retains a symmetric four-lobe structure around the OH_b_ at both 78 and 7 K. This indicates that OH_b_s are much weaker charge traps compared to bridging O vacancies (O_b_-vac). In addition, by sequentially removing the capping H of each OH_b_ using voltage pulses, we find that the annihilation of each OH_b_ is accompanied by the disappearance of some lobes in the filled state STM, thus verifying the direct correlation between OH_b_s and their excess electrons.

## Introduction

A *Polaron* is a quasiparticle formed when an electronic charge carrier introduced into a dielectric becomes localized at one of the symmetrically equivalent sites available. This alters the equilibrium positions of the surrounding lattice ions and subsequently creates a potential well that traps the carrier.^1^

These self-trapped polarons are believed to play a vital role in the physics and chemistry of many metal oxides, and technologically relevant phenomena as diverse as photolysis,^2^ high temperature conductivity^3^ and resistive switching.^4^ In light of this, polarons in materials including transition metal oxides, cuprates and 2D materials have been extensively characterized,^5−26^ and their influence on physical phenomena such as charge transport, surface reactivity and colossal magneto-resistance widely studied.^4,27−35^

**Figure 1:**
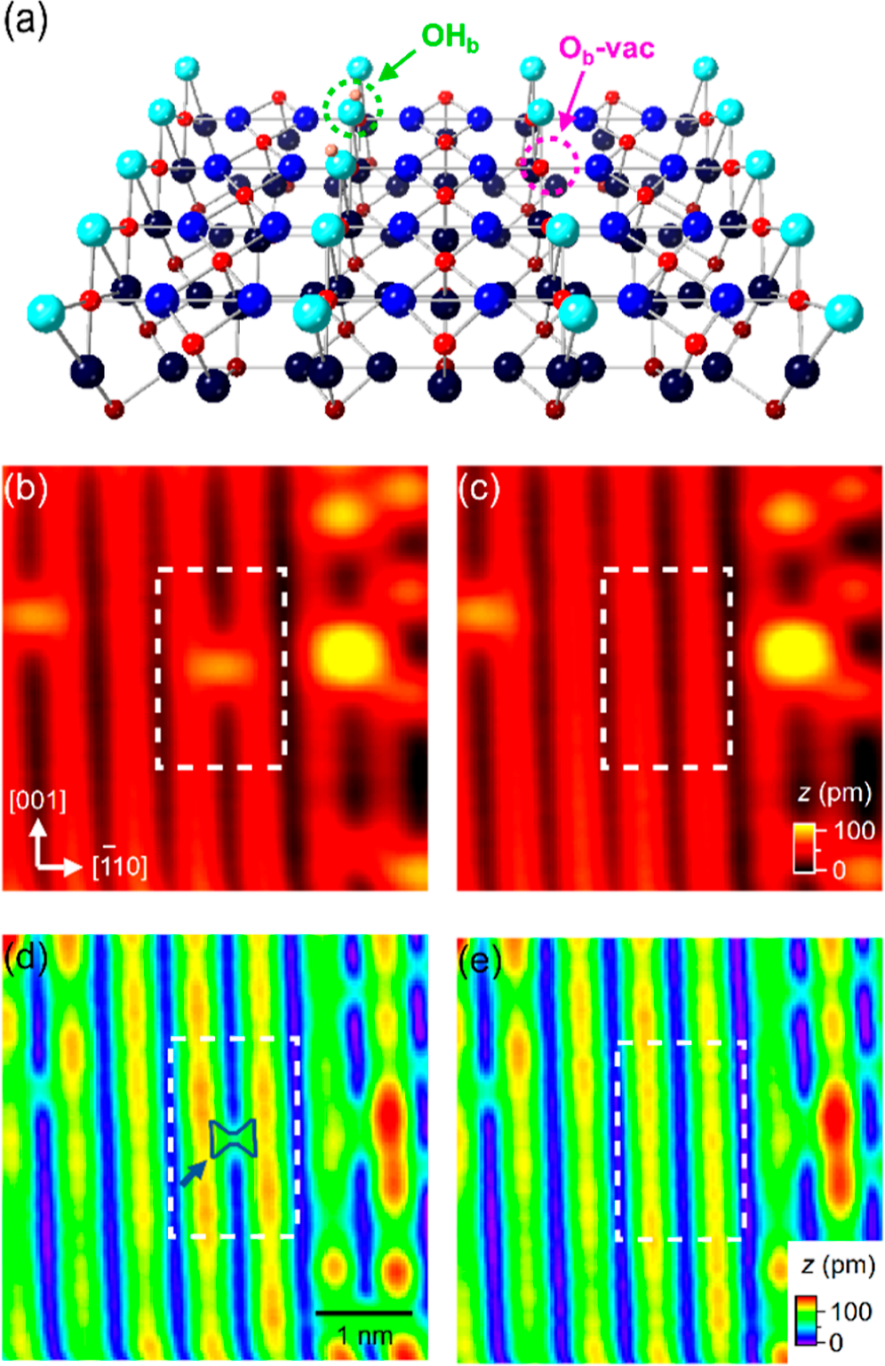
(a) Structural model of rutile TiO_2_(110). Spheres of different colors represent O_b_s (cyan), in-plane Os (mid blue), subsurface Os (dark blue), surface Ti ions (mid red), sub-surface Ti ions (dark red), and H atoms (pale pink), respectively. O_b_-vac and OH_b_ species are also indicated. (b) Empty- and (d) filled-states 78 K STM images of (4 nm)^2^ TiO_2_(110) with a single OH_b_. In (d), a bow-tie feature at the OH_b_ site that links the adjacent Ti_5c_ rows is indicated (c,e) As (b,d), recorded after removal of the capping H atom (marked by an arrow) from the OH_b_ with a 3 V, 1 ms tip pulse. Scan parameters (*V*, *I*): (b,d) 0.9 V, 50 pA, and (c,e) −1 V, 50 pA. Dashed rectangles mark the region where the four-lobe excess electron distribution surrounding the OH_b_ (d) disappears after the capping H removal.

Titanium dioxide (TiO_2_), a prototypical metal oxide system with applications ranging across heterogeneous catalysis, photolysis and solar cells etc.,^36−40^ has recently become a realistic material platform with which to study the polaron properties and their relevance to chemical processes. Taking the most stable (110) face of TiO_2_ in the rutile form as an example: its surface structure (Figure 1a) comprises rows of fivefold coordinated Ti^4+^ ions that alternate with those of twofold coordinated bridging O^2–^ ions (O_b_).^41^ TiO_2_ is a wide band-gap insulator (*E*_gap_ ∼ 3 eV), which can be made semiconducting upon reduction by cycles of ion sputtering and annealing.^42^ Such a reduction process leads to the formation of bridging oxygen vacancies (O_b_-vacs) on the surface,^43−45^ and two excess electrons for each created O_b_-vac. Previous studies showed that these excess electrons mainly reside at the subsurface Ti_6c_ sites (beneath the surface Ti_5c_ rows) surrounding the O_b_-vacs and reduce the associating Ti ions,^30,46−49^ with a small number occupying the surface Ti sites as observed by resonant photoemission diffraction.^50,51^ This results in Ti^3+^ 3d derived defect states, namely the band gap state (BGS), formed at ∼1 eV below the Fermi level (*E*_F_) within the band gap.^52,53^ Further studies verified the polaronic character of the O_b_-vac bound excess electrons,^54−56^ and their strong interaction with adsorbates in model chemical processes.^31,57,58^

O_b_-vacs on TiO_2_(110) are the most reactive sites for a diverse set of chemical reactions. Taking H_2_O adsorption on TiO_2_(110) as an example: H_2_O molecules adsorb dissociatively at O_b_-vacs, forming a pair of bridging hydroxyls (OH_b_) for each O_b_-vac.^59−63^ Over time, the OH_b_s within the OH_b_ pair diffuse away from each other and form two single OH_b_s. Previous studies showed that dissociative H_2_O adsorption on TiO_2_(110) does not cause any change to the BGS population. On this basis, one can assume that upon dissociative H_2_O adsorption, the excess electrons originally belonging to O_b_-vacs are transferred to the newly formed OH_b_ pairs, with each pair sharing two excess electrons. Also, it is believed that further splitting of a OH_b_ pair into two single OH_b_s should lead to a redistribution of the excess electrons between the two OH_b_s.

Scanning tunneling microscopy (STM), resonant photoemission diffraction, and density functional theory (DFT) calculations have been widely used to study the excess electron distribution in different metal oxide systems owing to their complementary advantages. In particular, using simultaneously recorded empty-(ES) and filled-states (FS) imaging (or dual-mode imaging) in STM, we previously observed that the O_b_-vac bound electron polaron distribution adopts a symmetric four-lobe structure surrounding the O_b_-vac at 78 K, which transforms into one of the three in-equivalent two-lobe structures as the temperature drops to 7 K.^56^ Here, we use low temperature dual-mode imaging in STM to determine how the bound polarons are distributed around the OH_b_ species following dissociative H_2_O adsorption. Moreover, we investigate their temperature dependent behavior. The answers to these questions will further our understanding of the intrinsic difference between O_b_-vac and OH_b_ species as charge traps. It will also illuminate the debate about the difference between the two types of OH_b_ (one formed at the O_b_-vac site and another at one of the neighboring O_b_ site).^60^

## Experimental Section

STM experiments were performed using an Omicron GmbH low temperature scanning tunneling microscope housed in an ultrahigh vacuum chamber with a base pressure in the 10–11 mbar region. To probe excess electrons associated with OH_b_, we performed simultaneously recorded filled (FS, using negative samples bias) and empty states (ES, using positive sample bias) STM imaging (namely, dual-mode imaging): in the forward scan along the fast scan direction, a line of topography data is recorded at positive sample bias; in the backward scan, a line of data is recorded with negative sample bias so that two images (ES and FS images) are recorded quasi-simultaneously. This eliminates the effects of thermal or piezo drift so that images obtained at opposite polarities can be directly correlated. To rule out the possibility of introducing any artifact from the forward scan to the backward scan, the polarity was occasionally reversed, i.e. the forward scans were negatively biased and backward scans were positively biased. No difference was observed in the resulting images.

To obtain a TiO_2_(110) single crystal sample with sufficient electrical conductivity for STM measurements at very low temperatures (*T* ∼ 7 K), we employed a special sample preparation procedure: first, a fresh rutile TiO_2_(110) sample (Pi-Kem) was subjected to about a hundred cycles of argon ion sputtering and vacuum annealing up to 1000 K; then, the as-prepared sample was left in the preparation chamber at a base pressure of 2 × 10^–10^ mbar at room temperature. In this environment, water from the residual vacuum reacts with O_b_-vacs on the sample surface, forming two OH_b_s for each O_b_-vac.^59−61^ In this way, a fully hydroxylated surface (h-TiO_2_) with a high density of OH_b_s is formed. This reaction removes all the surface O_b_-vacs.^64^

We previously showed that the O_b_-vac bound polarons separated from each other by at least three unit cells along the [001] direction, or at least one unit cell along [1̅10] have no measurable interaction with each other.^56^ On this basis, we prepared single OH_b_s on h-TiO_2_ as follows: first using dual-mode imaging to locate the OH_b_s isolated from regions of charged impurities. Then, using voltage pulses (3 V, 1 ms at 78 K; 3.5 V, 1 ms at 7 K) we removed the capping Hs of all other OH_b_s surrounding our targeted OH_b_ species.^65^ This led to a small surface area, usually about (5 nm)-2 containing only a few single noninteracting OH_b_s with their associated excess electron distributions.

## Results and Discussion

Figure 1 shows a dual-mode 78 K image of a single OH_b_ on TiO_2_(110) (Figure 1b,d), and those taken after the removal of its capping H (Figure 1c,e) using a +3 V, 1 ms tip pulse. Before the capping H removal, the FS image of a single OH_b_ is characterized by a bowtie-shaped feature at its position linking the neighboring Ti_5c_ rows, altogether with a nearly symmetric four-lobe structure with lobes located at the diagonal Ti_5c_ sites (Figure 1d). All of these features disappear after the capping H is removed (Figure 1e). Previous STM work by Minato et al. observed a similar FS image of single OH_b_.^52^ Previous DFT calculations of the hydroxylated TiO_2_(110) surface show that the Ti_6c_ sites in the second subsurface layer beneath the surface Ti_5c_ rows are the most stable sites for the OH_b_-polaron occupation.^46,66^ On this basis, we attribute the observed enhanced contrast along the Ti_5c_ rows in the FS images to the excess electrons populating in the second subsurface layer underneath the surface Ti_5c_ rows.

**Figure 2:**
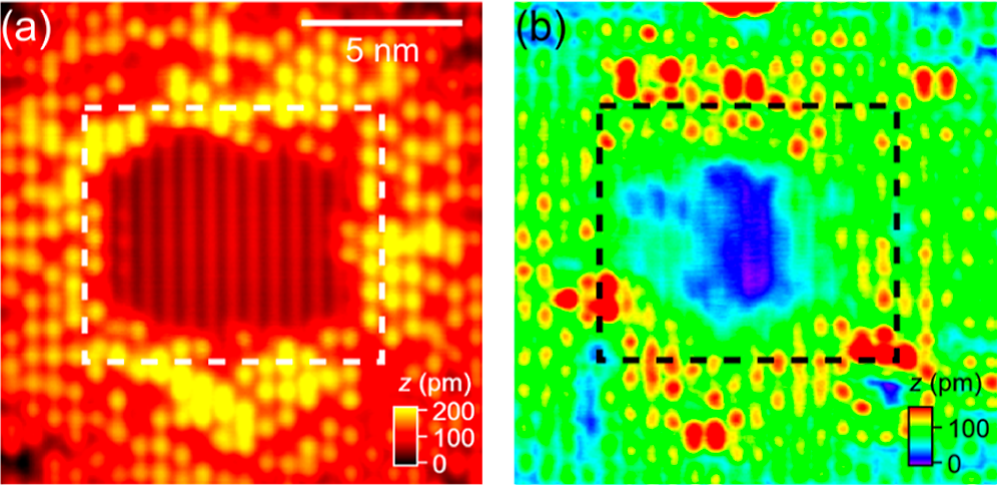
(a) ES- and (b) FS- images of h-TiO_2_ recorded after sequential removal of the capping Hs of all OH_b_s in the central part of the scanned region using +3.5 V, 1 ms tip pulses. The images were recorded at 7 K. Image size: (15 nm)^2^. Scan parameters (*V*, *I*): (a) +2 V, 10 pA; (b) −2 V, 1 pA.

We previously reported that the spatial distribution of the O_b_-vac bound excess electrons on the reduced surface of TiO_2_(110) (r-TiO_2_) transforms from a symmetric four lobe structure at 78 K into one of three asymmetric, two lobe structures at 7 K.^56^ Our findings confirmed the polaronic nature of the excess electrons on TiO_2_(110), which motivates this study of the spatial distribution of the OH_b_ bound excess electrons and its temperature dependence. Before looking into this, we first examined how the FS image contrast changes when the capping Hs of a group of OH_b_s are removed by using tip pulses. The results are shown in Figure 2, where all of the capping Hs at the center of the imaged region (marked by dashed rectangles) are removed (Figure 2a). In the FS images (Figure 2b), the H-stripped area appears much darker along the Ti_5c_ rows compared with the H-capped region. Hence, there is a direct correlation between OH_b_s and the excess electrons that appear as lobes on the Ti_5c_ rows in the FS images (Figure 2b). There are two likely modes in which the excess electrons could dissipate, depending on whether H is desorbed as a cation or a neutral species. In the former, electrons would be lost to the STM apparatus, while in the latter, the electrons would be captured by the bridging O ions.

**Figure 3:**
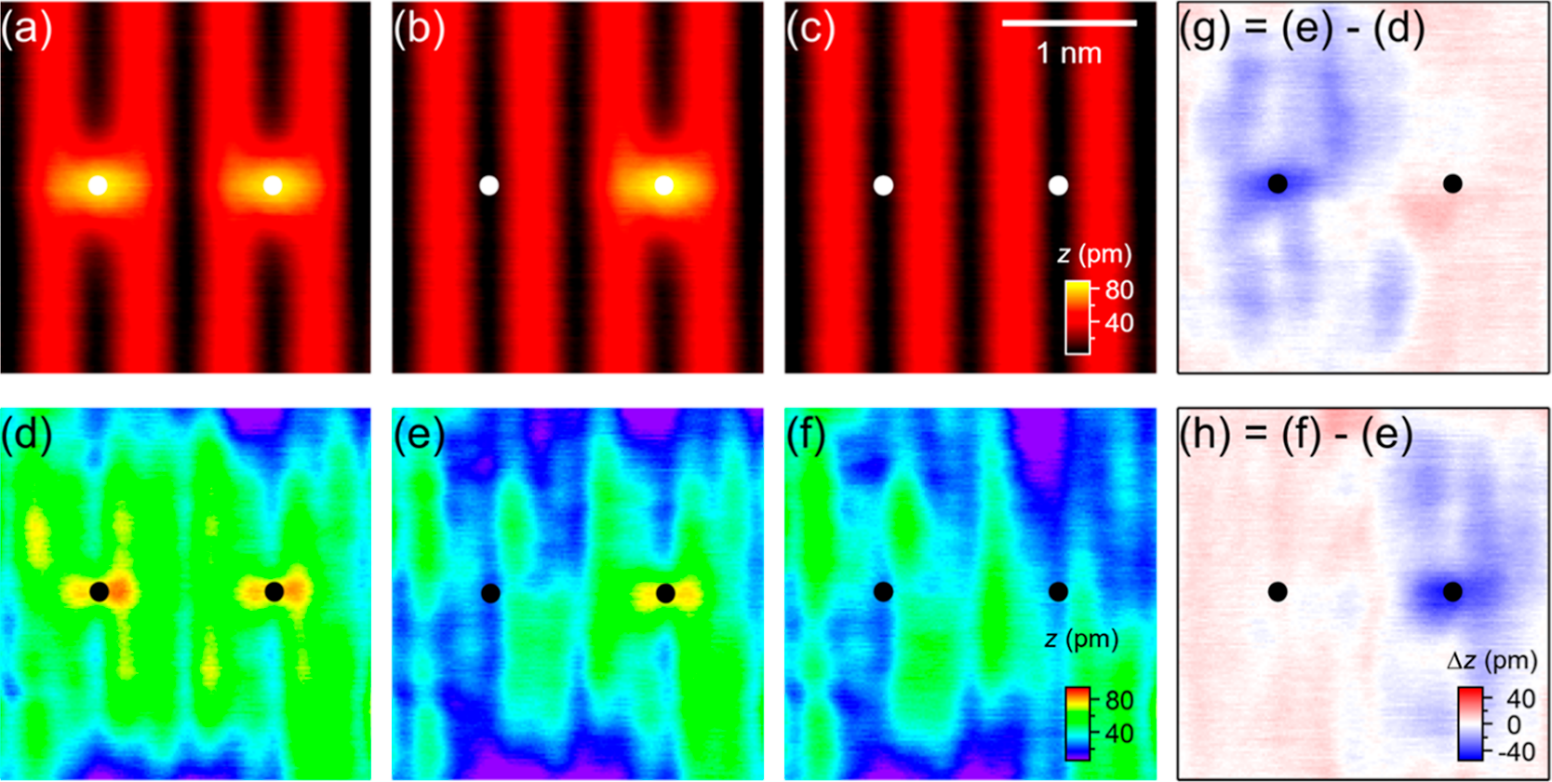
Simultaneously recorded (a) ES and (d) FS images of TiO_2_(110) containing two single OH_b_s. The images were recorded at 7 K. (b,e) As (a,d) recorded after the capping H of the OH_b_ on the left was removed by a +3.5 V, 1 ms tip pulse. (c,f) As (b,e) recorded after removal of the capping H of the OH_b_ on the right. Circles mark the OH_b_ positions. Image size: (2.74 nm)^2^. Scan parameters (*V*, *I*): (a–c) +2 V, 30 pA; (d–f) −2 V, 1 pA. (g–h) Difference images formed by subtraction of the FS image in (d) from that in (e), and of the FS image in (e) from that in (f), respectively.

Having established the relationship between OH_b_s and their associating excess electrons, we turn to the 7 K distribution of excess electrons surrounding single OH_b_ species. Figure 3 shows the dual-mode images recorded before and after the capping Hs of two single OH_b_s was sequentially removed using +3.5 V, 1 ms tip pulses. The ES images in Figure 3a–c simply evidence the conversion of OH_b_ to O_b_. In the FS image (Figure 3d), each OH_b_ image appears to be characterized by a nearly symmetric four-lobe structure. This is very different from the behavior of O_b_-vacs, the excess electron distribution of which is highly asymmetric at 7 K.^56^ We attribute this difference to the absence of polaron hopping at low temperature in the case of the O_b_-vac bound electrons but not for the OH_b_ polarons. Intuitively, the much faster hopping of the OH_b_ bound electrons evidenced at 7 K can be understood by (i) the weaker attractive force of OH_b_ (formal charge of 1+) to electrons as compared to O_b_-vac (formal charge of 2+), and (ii) the much smaller local distortion of the lattice from the formation of an OH_b_ by adding a H to an O_b_ as compared to that of O_b_-vac (by losing an O_b_).

Not only is there little difference between the spatial distribution of the OH_b_ bound excess electrons at 78 and 7 K, but there is also a similar effect of removing capping H at the two temperatures. Figure 3 shows the dual-mode images of two separated single OH_b_s, and those recorded after the sequential removal of their capping Hs by tip pulses. After the capping H on the left is removed (Figure 3b), not only the bowtie-shaped feature in the FS STM at the OH_b_ center disappears, the lobes distributed at the Ti_5c_ sites around the OH_b_ (Figure 3d) also vanish in the FS image (Figure 3e). The similar observation also applies to the OH_b_ on the right (see Figure 3e,f). To better visualize the changes in the FS images, we present in Figure 3g,h, the difference images formed by subtraction of the FS images taken before and after each capping H removal. There, one clearly can see that each OH_b_ is characterized by a bowtie-shaped feature at the center with four lobes distributed at each of the second nearest Ti_5c_ sites around it. This again confirms the observation of a nearly symmetric, four-lobe structure for the distribution of the OH_b_-bound excess electrons at 7 K. Taking a closer look at the difference image (Figure 3g), we also observe a redistribution of the excess electrons in the vicinity of the OH_b_ on the right after the capping H of the OH_b_ on the left is removed, as evidenced by the additional lobe of density loss in the bottom region between the two OH_b_s. In addition, the difference images (Figure 3g,h) show only a reduction in the FS contrast in close proximity to the OH_b_s, while that in the surrounding region remains unchanged. This is consistent with dissipation of the excess electrons through the STM apparatus or capture by bridging O ions, as noted above.

Previous studies showed that when a H_2_O molecule adsorbs dissociatively at an O_b_-vac, two OH_b_s, one at the O_b_-vac site (namely v-OH_b_) and another at one of the two nearest-neighboring O_b_ ions (namely b-OH_b_), are formed.^59,62^ A later STM study by Zhang et al. determined that the capping Hs of b-OH_b_s are ten times more likely to hop along the Ti_5c_ rows compared to v-OH_b_s, evidencing their inequivalence.^60^ One possible scenario is that the distribution of the excess electrons, originally belonging to the O_b_-vac, between v- and b-OH_b_ within a newly formed OH_b_ pair is uneven. To gain further insight into this, we employed a “pulse and track” approach, i.e. recorded dual-mode STM images before and after each successive removal of the capping Hs from the OH_b_s within the OH_b_ pair using tip pulses. In doing so, we aim to find out how the excess electrons are trapped and how they are distributed around each of the OH_b_s within the OH_b_ pair. Before discussing that, we first discuss how successive tip-induced removal of the capping Hs of the OH_b_ species surrounding a OH_b_ pair influence the polaron distribution of the OH_b_ pair.

**Figure 4:**
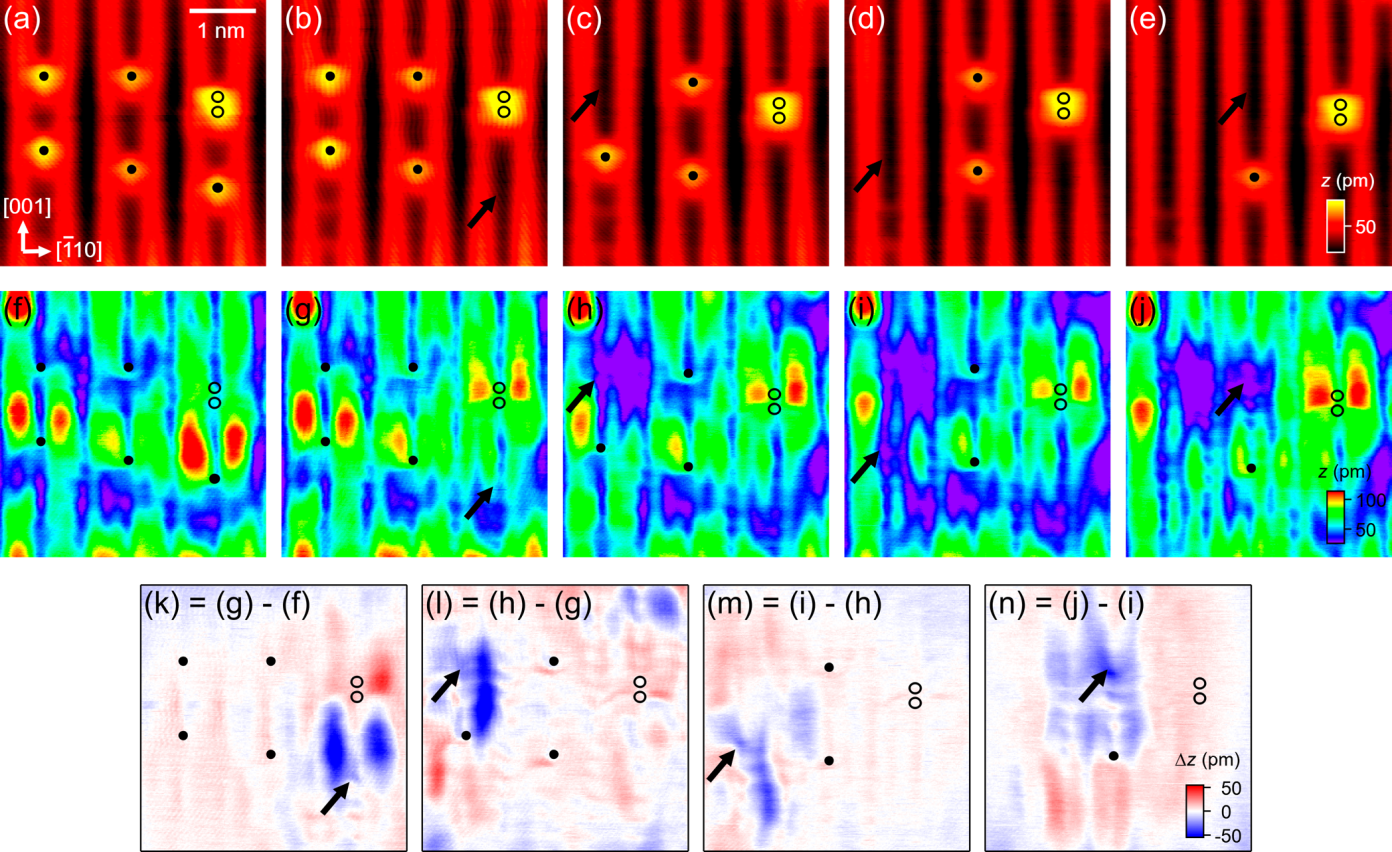
Simultaneously recorded (a) ES and (f) FS images of h-TiO_2_. Before imaging, the capping Hs of most OH_b_s originally present were removed using +3.5 V, 1 ms tip pulses, leaving only five OH_b_s and one OH_b_ pair remaining in the imaged region. (b–j) As (a–f) following the sequential removal of the capping H of each of the OH_b_ species using the same tip pulses. Solid circles mark the positions of single OH_b_s. Open circles mark those in the OH_b_ pair. Arrows indicate the capping H being removed in each frame. All images were recorded at 6.6 K. Image size: (4 × 4) nm^2^. Scan parameters (*V*, *I*): (a–e) +2 V, 30 pA; (f–j) −2 V, 1 pA. (k–n) Difference images formed by subtraction of the FS images obtained before and after the removal of the capping H within each OH_b_ species.

Figure 4 shows a series of simultaneously recorded dual-mode images, recorded at 6.6 K, taken before and after the sequential removal of each of the capping Hs with tip pulses (+3.5 V, 1 ms). Before imaging, the capping Hs of most of the OH_b_s originally present in the scanned region were removed using the same tip pulses. This leaves only five OH_b_s and one OH_b_ pair remaining in the scanned region. As shown in the FS images (Figure 4f–j) and in the difference images (Figure 4k–n), the removal of each capping H is always accompanied by changes in image contrast in the FS image. Taking the OH_b_ at the bottom right as an example, after its capping H is removed (Figure 4b), the two lobes originally present at the Ti_5c_ sites above that of OH_b_ disappear (Figure 4f). Their disappearance is also accompanied by some increase in the intensity of the lobes at the Ti sites above the OH_b_ pair at the top right of the image (Figure 4g). This indicates that not only does the removal of that capping H lead to a dissipation of the associated excess electrons but also it results in modification of the excess electron distribution surrounding that OH_b_-pair. Similar changes to the excess electron distributions surrounding the OH_b_ and OH_b_ pair on the surface have also been observed following the removal of other capping Hs, see the images (Figure 4g–j) and the corresponding difference images (Figure 4l–n) for such changes.

**Figure 5:**
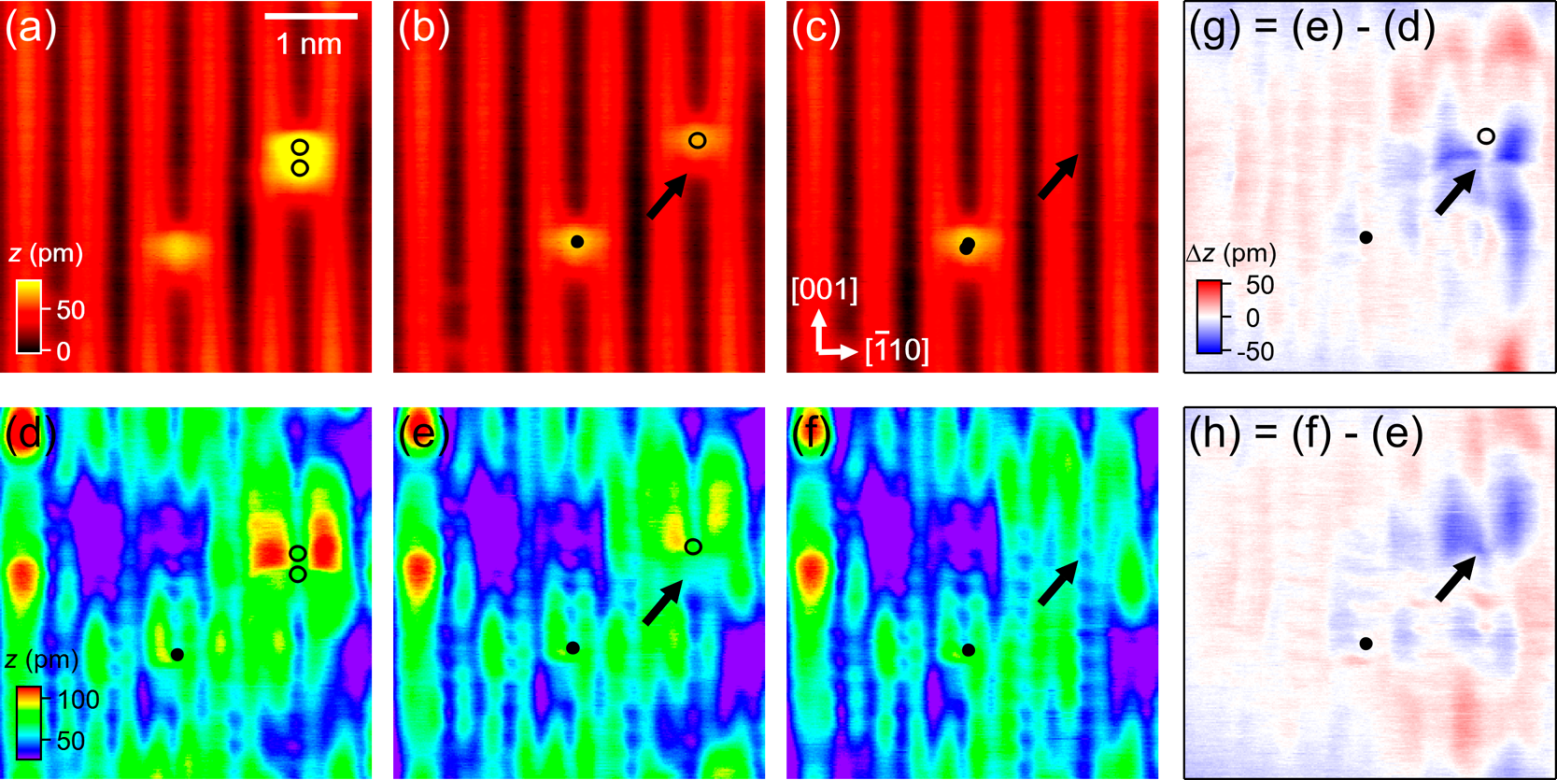
Simultaneously recorded (a) ES and (d) FS images of TiO_2_(110) containing one single OH_b_ and one OH_b_ pair. (b–c) As (a), but recorded after the capping H atoms of the OH_b_ pair were removed sequentially using +2.6 V, 200 ms tip pulses. Solid circles mark the positions of single OH_b_. Open circles mark the OH_b_ within the OH_b_ pair. Arrows indicate the capping H that was removed in each frame. (e–f) Corresponding FS images of (b–c), respectively. All images were recorded at 6.6 K. Image size: (4 × 4) nm^2^. Scan parameters (*V*, *I*): (a–c) ±2 V, 30 pA; (d–f) −2 V, 1 pA. (g–h) Difference images formed by subtraction of the FS images obtained before and after the removal of each capping H within the OH_b_ pair.

In addition to studying the influence of neighboring OH_b_s on the polaron distribution surrounding a OH_b_ pair, we have also investigated how the polaron distribution surrounding an OH_b_ pair changes upon the sequential removal of its capping Hs, and our results are shown in Figure 5. The initial empty and filled state images are shown in Figure 5a,d, respectively. The filled state image evidences a distribution of the OH_b_ pair bound polarons that has a three lobe structure. The apparent asymmetry in the FS image is consistent with the asymmetric behavior observed by Zhang *et al.* in the mobility of the two types of bridging hydroxyls.^60^ After the first capping H within the OH_b_ pair was removed by a tip pulse (Figure 5b), the lobes become significantly weaker in intensity and displace away from their original positions (Figure 5e,g). Then, after the second capping H was removed (Figure 5c), the lobes further weaken and dissipate further away from the original position of the OH_b_ pair (Figure 5f,h). Based on the above, we conclude that, first, OH_b_s are weaker as charge traps compared to a OH_b_ pair, and second, as all the charge traps on the surface are removed, the excess electrons originally bound to those charge traps are dissipated. Meanwhile, the resulting absence of any charge traps leads to much more uniform appearance along the Ti_5c_ rows (Figure 5f).

Through comparison of the STM data shown in Figures 3–5, we find that the almost symmetric four-lobe structure of the spatial distribution of the OH_b_ bound polarons at *T* = 7 K transforms into one of the asymmetric two- or three-lobe structures as temperature is reduced to 6.6 K. We attribute such change in the polaronic distribution to the temperature-dependent hopping behavior of polarons: at 7 K polarons still hop between the subsurface Ti_6c_ sites surrounding a OH_b_ and their motion starts to freeze; at 6.6 K their motion becomes completely frozen and depending on the local chemical environment,^56^ their distribution about a OH_b_ adopts one of the asymmetric structures.

## Summary

To summarize, employing dual-mode imaging to study the spatial distribution of the OH_b_ bound excess electrons on the (110) surface of TiO_2_ rutile, we found that their distributions retain a symmetric, four-lobe structure at temperature of 7 K, suggesting that OH_b_s are much weaker as charge traps compared to O_b_-vacs, with their associated polarons requiring much less energy to hop over different Ti sites surrounding the vacancies. In addition, using voltage pulses to sequentially remove the capping H of each of the OH_b_s and monitoring the corresponding changes in the image contrast within the FS STM, we found that every capping H removal is accompanied by the disappearance of some FS contrast surrounding the removed capping H position, thus verifying that each OH_b_, once formed, is accompanied by a polaron.
